# Evaluation of the management of high-risk obstetric patients living with epilepsy: A retrospective study from KwaZulu-Natal, South Africa

**DOI:** 10.4102/safp.v68i2.6223

**Published:** 2026-02-21

**Authors:** Sumeshni Birbal, Frasia Oosthuizen, Urisha Naidoo

**Affiliations:** 1 Department of Health Sciences, Faculty of Pharmaceutical Sciences, University of KwaZulu-Natal, Durban, South Africa

**Keywords:** epilepsy, pregnancy, anti-epileptic drugs, teratogenic, malformations

## Abstract

**Background:**

Epilepsy affects approximately 60 million individuals worldwide, with women comprising nearly 50% of the cases. Physiological changes during pregnancy can alter anti-epileptic drug (AED) pharmacokinetics and seizure thresholds, necessitating close therapeutic drug monitoring (TDM). Uncontrolled maternal seizures increase the risk of maternal morbidity, foetal hypoxia and miscarriage. Several AEDs have a known teratogenic potential, highlighting the importance of adherence to clinical guidelines to optimise maternal and foetal outcomes.

**Methods:**

A retrospective quantitative study was conducted at a high-risk obstetrics clinic in a public tertiary hospital in KwaZulu-Natal, South Africa, using clinical records from a 5-year period. Of 131 cases, *N* = 90 met the inclusion criteria. Data were extracted into Microsoft Excel^®^ and analysed using SPSS^®^ (v29). Descriptive and inferential analyses (chi-square, Fisher’s exact, Spearman’s correlation, Mann–Whitney U and logistic regression) were used to assess the associations between variables.

**Results:**

Sodium valproate was prescribed to 60% of patients in the cohort. Folic acid supplementation was documented in 70% of cases (*n* = 63). Sodium valproate use was significantly associated with polytherapy (*p* = 0.007). Therapeutic drug monitoring was performed in 56.7% of patients and prenatal ultrasounds in 67.8%. A strong correlation was observed between the total admission frequency and seizure-related admissions (Spearman’s ρ = 0.74, *p* < 0.001).

**Conclusion:**

The study identified gaps in care, including suboptimal AED selection, limited TDM, inconsistent ultrasound use, frequent polytherapy and increased admissions from poor seizure control. Addressing these may improve the safety and effectiveness of epilepsy management in pregnancy.

**Contribution:**

This study contributes novel, context-specific evidence on real-world epilepsy management during pregnancy. It identifies critical gaps in clinical practice, AED selection and therapeutic monitoring.

## Introduction

Epilepsy remains a significant non-communicable disease with global health relevance.^1^ It is defined as a chronic disorder of the brain characterised by an enduring disposition towards recurrent unprovoked seizures and is recognised as the fourth most common neurological disorder in the world.^1^

Epidemiology estimates indicate that between 4 and 10 per 1000 people worldwide suffer from epilepsy.^2^ The World Health Organization (WHO) estimates that 25 million people in Africa suffer from active epilepsy, with two-thirds living in low- and middle-income countries (LMICs).^3^ Despite this significant epidemiological burden, the management of epilepsy in Africa has remained inadequate, with the African population having the highest associated morbidity and mortality.^2^ In LMICs, patients have to endure a reduced quality of life, heightened risks of adverse health outcomes and premature mortality, often because of the country’s developmental and economic status.^4,5^ First-line anti-epileptic drugs (AEDs) cause adverse effects in nearly half of the patients undergoing treatment, while drug resistance is observed in 32% of these patients.^5^

Globally, at least 15 million of the total population living with epilepsy are women of child-bearing age.^6^ The WHO defines a woman’s reproductive age as 15–49 years old.^3^ In sub-Saharan Africa, the peak prevalence of epilepsy occurs in the 20–29 age group for both men and women, resulting in a significant proportion of women with epilepsy (WWE) who are of childbearing age.^4^

There is an elevated risk of severe maternal and perinatal complications faced by WWE, including eclampsia, placental abruption, embolism, cardiac disorders, complications arising from obstetric procedures and severe antepartum or postpartum haemorrhage, as well as foetal hypoxia, neural tube defects (NTDs), preterm delivery, low birth weight and major congenital malformations (MCMs).^7^

The risk regarding the teratogenic potential of AEDs has been a concern that dates back over 50 years, following early reports of six children who were born with a cleft lip and palate because of AED exposure *in-utero*.^6^ Numerous subsequent studies have confirmed an increased prevalence of MCMs and adverse neurodevelopmental outcomes in children born to WWE, which was mainly correlated to foetal AED exposure.^8,9,10,11^

The risk of major birth defects is estimated at 10-11% in intrauterine AED exposure, *in-utero*, compared to 2-3% in the general population.^10,11^ Common MCMs include NTDs (e.g. *spina bifida*), craniofacial abnormalities, as well as cardiac and limb defects.^10,11^ Severe abnormalities that will require treatment after birth or lead to long-term health problems include malformations of the spinal cord and heart, including cleft lip and palate.^12^ Other minor birth defects that primarily affect the baby’s appearance (dysmorphic features) are facial abnormalities (e.g. eyes set too wide apart, a short upper lip) and small fingers and toes with nail dystrophy.^13,14,15,16^ Beyond structural malformations, neurodevelopmental disorders occur in 30-40% of AED exposed children who later experience cognitive impairment, lower IQ levels and developmental delay.^10,11^ The risk of autism spectrum disorder (ASD) is 2–5 times higher in this group with additional associations to attention-deficit hyperactivity disorder (ADHD) and social communication difficulties.^10,11^ Foetal valproate syndrome (FVS) represents a severe outcome of AED exposure, manifesting as facial dysmorphism (e.g. epicanthal folds, broad nasal bridge) as well as growth retardation, skeletal abnormalities and delayed speech and motor skills.^10,11^

The high risk of foetal anomalies associated with AED use necessitates the routine application of prenatal ultrasound assessments to monitor foetal development.^13,14^ Systematic ultrasonographic evaluation allows for the early detection of structural malformations, including both MCMs and minor dysmorphic features.^13,14^

There are many concerns surrounding WWE and safe pregnancies^17^ The intake of folic acid throughout the first trimester of pregnancy provides a supportive environment for neural tube formation and decreases the likelihood of developing a NTDs when using AEDs such as sodium valproate.^18,19^ Decades of research have established that AEDs show varying degrees of teratogenic risk.^16^ Sodium valproate has been identified as having the highest potential for causing MCMs and neurodevelopmental problems when compared to other AEDs.^7^

Seizure control is crucial for maternal health and foetal well-being, yet it is often compromised by a decline in serum concentrations of certain AEDs during pregnancy.^18^ Routine therapeutic drug monitoring (TDM) during pregnancy is necessary to confirm that serum AED concentrations remain within therapeutic ranges since physiological changes, such as increased plasma volume and alterations in renal function, can have significant effects on drug metabolism and serum AED levels.^19^ Therapeutic drug monitoring therefore assists in optimising dosing and minimising the risks of teratogenicity and other adverse drug effects.^19^ Furthermore, for women taking enzyme-inducing AEDs such as phenytoin, phenobarbitone and carbamazepine, there is an increased risk of vitamin K deficiency, which can result in bleeding complications in the newborn.^20^ The administration of antepartum vitamin K is therefore recommended to reduce the risk of neonatal haemorrhage and support coagulation pathways, ensuring better outcomes for both mother and baby.^19^

According to the 130 million annual global births and the estimate that WWE represent 0.5% of those births, it is estimated that each year 650 000 infants will be born to WWE worldwide.^6^ Therefore, pregnancy safety for WWE is not just a priority for the individual woman, but also a major public health concern.^6^ Addressing these challenges requires a coordinated approach across all levels of the healthcare system and the active involvement of multidisciplinary teams.^6^ Primary care clinicians are often the first point of contact and play a pivotal role in preconception counselling, rational drug use and ensuring supplementation and monitoring are conducted prior to referral.^6^ Nurses and midwives provide critical support by providing antenatal education, adherence support and routine screening, while pharmacists contribute through prescription review, counselling patients on teratogenic risks, ensuring access to safer alternatives and reinforcing the importance of folic acid supplementation.^6^ At the same time, district-level clinicians often contribute to local clinical governance and policy implementation, positioning them to advocate for safer prescribing practices, standardised supplementation protocols and improved referral pathways for WWE.^6^ Accordingly, the aim of this study was to critically evaluate the management of high-risk obstetric patients living with epilepsy at a tertiary hospital in KwaZulu-Natal (KZN), South Africa (SA), and to compare current practices with established international clinical guidelines.

## Research methods and design

A retrospective quantitative study was carried out using patient clinical data/records from a high-risk obstetrics clinic within a public tertiary hospital located in KZN, SA. This setting was selected because it is the only public sector facility in the province where patients with complex maternal conditions, including high-risk obstetric patients are referred for specialised tertiary-level care. Patient records are electronically stored in a standardised database, ensuring consistency in data collection parameters across cases and enhancing the reliability of results, thereby providing a sufficiently large patient population for meaningful analysis.

A convenience sampling method was used and included all pregnant patients diagnosed with epilepsy (verified by the inclusion/exclusion criteria) that attended the clinic from 01 January 2018 to 31 December 2022. The study sample was identified from patients automatically documented on the hospital’s electronic database and consisted of all available and eligible cases during the study period. The patient data were selected using the international classification of diseases (ICD-10) codes – a standardised system used to translate diagnoses of diseases and medical conditions from words into alphanumeric codes that permit easy storage, retrieval and analysis of data.^20^ The ICD-10 code O99.3 (mental disorders and diseases of the nervous system complicating pregnancy, childbirth and the puerperium) was used to extract the data. In addition, either of the following ICD1-10 codes were also used:

- G40.0: Localisation-related (focal) (partial) idiopathic epilepsy and epileptic syndromes with seizures of localised onset
- G40.1: Localisation-related (focal) (partial) symptomatic epilepsy and epileptic syndromes with simple partial seizures
- G40.2: Localisation-related (focal) (partial) symptomatic epilepsy and epileptic syndromes with complex partial seizures
- G40.3: Generalised idiopathic epilepsy and epileptic syndromes
- G40.4: Other generalised epilepsy and epileptic syndromes
- G40.5: Special epileptic syndromes
- G40.6: Grand mal seizures, unspecified (with or without petit mal)
- G40.7: Petit mal, unspecified, without grand mal seizures
- G40.8: Other epilepsy
- G40.9: Epilepsy, unspecified

Extracted data were received electronically in the form of case reports as a Microsoft Excel^®^ document from the hospital’s MediTech^®^ system (electronic database of patient clinical information). No identifiable patient information was used in the study, thereby ensuring confidentiality. The case reports included variables pertaining to patient demographics as well as medicine and management history.

A total of 131 case reports were received, of which 41 were excluded from the study as per the inclusion and exclusion criteria below:

Inclusion criteria:

- Pregnant patients living with epilepsy that were treated at the institution during the period 01 January 2018 to 31 December 2022
- ICD-10 codes that were used for the above patients upon diagnosis had to include O99.3 and either of the following: G40.0, G40.1, G40.2, G40.3, G40.4, G40.5, G40.6, G40.7, G40.8, G40.9
- Inpatient or outpatient

Exclusion criteria:

- Women without a confirmed diagnosis of epilepsy
- Women with epilepsy who were not pregnant during the study period
- Duplicate records representing the same patient
- Records with incomplete essential material for multiple variables

Therefore, the sample size (*N*) accumulated to 90 case reports for analysis. The data were coded using numbers and thereafter transcribed onto a predesigned data extraction tool, using Microsoft Excel^®^, and analysed using SPSS (version 29). Missing values were assessed for each variable and were coded as ‘unknown’ and included in the analysis where relevant. For cross-tabulations, chi-square and correlations, the cases missing the particular variable being measured were excluded. For regression analyses, only cases with complete data regarding the variables being measured were included. The primary outcome was to measure adherence to guideline recommendations regarding the management of epilepsy in pregnancy. The secondary outcomes included factors pertaining to the frequency and reasons behind hospital admissions and neonatal outcomes such as gestational age, birth weight and head circumference.

The following data were extracted:

- Demographics (age, ethnicity)
- Medication history (AEDs, folic acid, monotherapy, polytherapy)
- Ultrasound visits
- Serum AED levels (TDM)
- Hospital admission frequency
- Diagnosis during hospital admission(s)
- Laboratory tests
- Gestational age
- Birth weigh
- Head circumference

Descriptive statistics, nonparametric tests and regression analyses were used to evaluate patient demographics, AED use and maternal and neonatal outcomes. Measures of central tendency (mean, median and mode) were used to summarise continuous variables such as gestational age, head circumference and birth weight. Inferential statistical tests were employed to assess associations and differences between variables.

Relationships between categorical variables were assessed using chi-square and Fisher’s exact tests. For continuous variables not normally distributed, nonparametric tests such as the Mann–Whitney U and Spearman’s rank correlation were used. Binary logistic regression was conducted to assess the association between variables (sodium valproate, folic acid, gestational age at delivery, TDM, polytherapy, birth weight) and was guided by clinical relevance, prior evidence from literature and the potential effects on the outcome. Model fit and odds ratios (ORs) were reported with 95% confidence intervals. Statistical significance was defined as a *p*-value of less than 0.05 (*p* < 0.05).

### Ethical considerations

Full ethical approval for the study was obtained from the Biomedical Research Ethics Committee (BREC) at the University of KwaZulu-Natal (BREC/00004455/2022). Permission was also obtained from the high-risk obstetric unit head of department (HoD), the hospital’s chief executive officer (CEO) and the National Department of Health (NDoH) ethics committee (KZ_202210_029).

## Results

### Overview of patient demographics

A total of 90 (*N* = 90) case reports were analysed. The majority of patients were between 26 years and 30 years of age (*n* = 21, 23.3%), followed by equal proportions in the 21-25-years-old and 31-35-years-old age groups (each *n* = 19, 21.1%). The youngest patient was 16-years-old whereas the oldest was 44-years-old. The most frequently occurring age was 29 years, with a mean age of 28.48 ± 6.60 years. Most participants were of African descent (*n* = 79, 87.8%), with the remainder being Indian (*n* = 8, 8.9%) and White (*n* = 3, 3.3%).

### Analysis of anti-epileptic drugs, folic acid, vitamin K, monotherapy and polytherapy

The types of AEDs prescribed and their respective frequencies are presented in Table 1. Sodium valproate was the most commonly prescribed AED (*n* = 54, 60.0%), followed by carbamazepine (*n* = 25, 27.8%) and lamotrigine (*n* = 25, 27.8%). Topiramate and phenytoin were each used in 5.6% (*n* = 5) of cases and clonazepam in 8.9% (*n* = 8) of cases As shown in Table 2, folic acid supplementation (5 mg daily) was recorded for 70% of patients (*n* = 63).

**TABLE 1:** Distribution of anti-epileptic drug utilization across maternal age groups (*N* = 90).

Variable			Anti-epileptic drug													
Age (years)	*n*	%	Sodium valproate (*n* = 54)		Lamotrigine (*n* = 25)		Carbamazepine (*n* = 25)		Clonazepam (*n* = 8)		Phenytoin (*n* = 5)		Topiramate (*n* = 5)		Other (*n* = 4)	
			*n*	%	*n*	%	*n*	%	*n*	%	*n*	%	*n*	%	*n*	%
15–20	14	15.6	11	78.6	3	21.4	5	35.7	1	7.1	2	14.3	1	7.1	0	0
21–25	19	21.1	12	63.2	2	10.5	3	15.8	1	5.3	0	0	1	5.3	2	10.5
26–30	21	23.3	12	57.1	9	42.9	6	28.6	3	14.3	1	4.8	2	9.5	1	4.8
31–35	19	21.1	8	42.1	5	26.3	6	31.6	2	10.5	0	0	0	0	0	0
36–40	15	16.7	9	60.0	6	40.0	5	33.3	1	6.7	2	13.3	1	6.7	1	6.7
≥ 41	2	2.2	2	100	0	0	0	0	0	0	0	0	0	0	0	0

**TABLE 2:** Anti-epileptic drug utilization in relation to supplementation and monitoring practices (*N* = 90).

Variable			Anti-epileptic drugs													
Drug-related parameter	*n*	%	Sodium valproate		Lamotrigine		Carbamazepine		Clonazepam		Phenytoin		Topiramate		Other	
			*n*	%	*n*	%	*n*	%	*n*	%	*n*	%	*n*	%	*n*	%
Folic acid	63	70.0	34	54.0	20	31.7	19	30.2	7	11.1	4	6.4	5	7.9	4	6.4
Vitamin K	0	0	0	0	0	0	0	0	0	0	0	0	0	0	0	0
Monotherapy	45	50.0	25	55.6	7	15.6	13	28.9	0	0	0	0	0	0	0	0
Polytherapy	34	37.8	29	85.3	18	52.9	12	35.3	8	23.5	5	14.7	5	14.7	4	11.8
Therapeutic drug monitoring	51	56.7	39	76.5	15	29.4	12	23.5	6	11.8	4	7.8	3	5.9	3	5.9
Ultrasound screening	61	67.8	37	60.7	16	26.2	16	26.2	4	6.6	3	4.9	3	4.9	3	4.9

**FIGURE 1:**
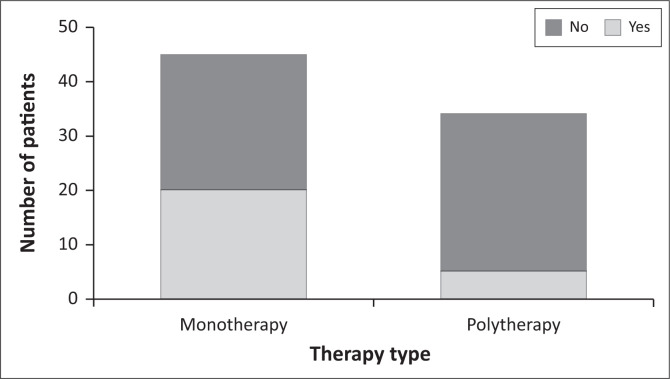
Sodium valproate use by therapy type (monotherapy vs. polytherapy). A higher proportion of patients on polytherapy were prescribed sodium valproate compared to those on monotherapy (OR = 0.22; 95% CI 0.07–0.66, Fisher’s exact test, *p* = 0.007).

Of the *n* = 54 patients on sodium valproate, *n* = 34 (63.0%) also received folic acid, while 37.0% (*n* = 20) of patients had no record of supplementing with folic acid (Table 2). Among those on folic acid (*n* = 63), *n* = 33 patients were on monotherapy and *n* = 11 patients on polytherapy. Monotherapy and polytherapy were prescribed to 50% (*n* = 45) and 37.8% (*n* = 34) of patients, respectively. A significant association was found between sodium valproate use and polytherapy (OR = 0.22; 95% CI 0.07-0.66, Fisher’s exact test, *p* = 0.007) (Figure 1). Notably, none of the patients received antepartum vitamin K (Table 2).

### Gestational age, therapeutic drug monitoring, ultrasounds, baby birth weight and head circumference

Neonatal outcomes, including gestational age, birth weight and head circumference, were extracted from delivery records where available. Birth data were only recorded for 50% (*n* = 45) of patients. Of these, 73.3% (*n* = 33) underwent caesarean delivery, while 26.7% (*n* = 12) delivered vaginally. The mean gestational age at delivery was 36.07 ± 5.99 weeks. Newborn head circumference was recorded for *n* = 41 patients, with the mean of 34.72 ± 3.57 cm. Birth weights were recorded for *n* = 42 newborns (46.7% of the cohort) with the mean birth weight of 2.80 ± 0.70 kg. Serum AED levels (TDM) were conducted in *n* = 51 (56.7%) of patients, while ultrasounds were performed for *n* = 61 (67.8%) of patients. Patients on sodium valproate were five times more likely to undergo TDM than those not on sodium valproate (OR = 5.20; 95% CI 2.09-12.97, *p* < 0.001). A statistically significant positive correlation was observed between gestational age and birth weight (Spearman’s *p* = 0.72, *p* < 0.001).

### Admission frequency and admission relation to seizures/epilepsy during pregnancy

**FIGURE 2:**
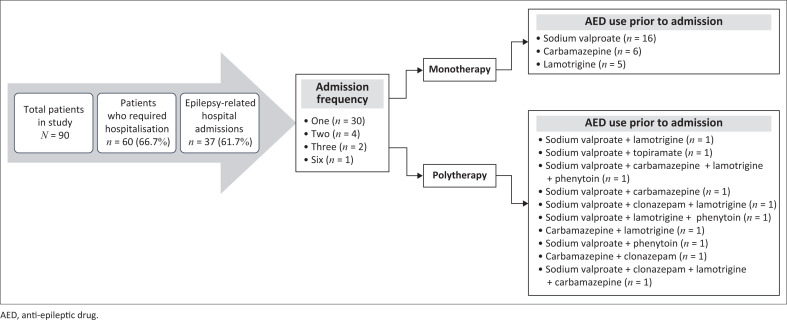
Anti-epileptic drug regimens and epilepsy-related admission patterns.

As illustrated in Figure 2, 66.7% of patients (*n* = 60) required hospital admission during pregnancy. Of these, 61.7% (*n* = 37) were admitted for seizure-related events. Notably, 20 of these admissions (40%) belonged to *n* = 7 patients requiring multiple hospitalisations. A strong correlation was observed between total admission frequency and seizure-related admissions (Spearman’s *p* = 0.74, *p* < 0.001). Figure 2 also presents the distribution of AED prescriptions among hospitalised patients. Sodium valproate was the most commonly prescribed AED (*n* = 24, 64.9%), followed by carbamazepine (*n* = 11, 29.7%), lamotrigine (*n* = 11, 29.7%), clonazepam (*n* = 3, 8.1%), phenytoin (*n* = 3, 8.1%) and topiramate (*n* = 1, 2.7%). Patients on sodium valproate were more likely to require hospital admission for seizure control during pregnancy (OR = 3.58; 95% CI 1.38-9.34, *p* = 0.006).

## Discussion

Managing epilepsy during pregnancy presents a complex challenge, requiring a careful balance between maternal seizure control and foetal safety.^9,11,13^ This study underscores the persistent difficulties in optimising epilepsy management in this population, and while this analysis reflects a tertiary hospital setting, the findings carry significant implications across all levels of care.^21,22,23^ Within the South African public healthcare context, we observed high rates of sodium valproate use, inadequate folic acid supplementation, frequent reliance on polytherapy, elevated caesarean section rates and suboptimal use of TDM. Several key findings emerge, emphasising both clinical and system-level challenges. The findings align with global research on epilepsy in pregnancy and highlight critical areas for clinical improvement.^24,25,26,27^

In this study, 60% of WWE were prescribed sodium valproate – a strikingly high proportion given its well-documented teratogenic risks.^24,25^ This mirrors findings from a study conducted over 3 years in the Western Cape, highlighting persistent reliance on sodium valproate across South African provinces.^10^ This is alarming given that the South African NDoH standard treatment guidelines (STGs) and the South African Health Products Regulatory Authority (SAHPRA) explicitly advises against the use of sodium valproate in pregnancy and in women of childbearing potential, unless no alternative is available.^10,28^ Globally, regulatory bodies – including the European Medicines Agency (EMA) and the UK Medicines and Healthcare Products Regulatory Agency (MHRA) have issued strong warnings and restrictions against sodium valproate use in pregnancy.^10,11^

In cases where sodium valproate is highly effective in maintaining seizure control and where access to safer alternatives (e.g. lamotrigine and levetiracetam) are limited in LMICs, its use may be justified.^29,30^ Additionally, if it is prescribed prior to conception, foetal exposure may occur before medication adjustments are made.^31^ Also, in established regimens in women who are controlled, clinicians may be reluctant to alter therapy.^24^ In these instances, the use of sodium valproate may be warranted following a rigorous risk–benefit assessment, accompanied by close clinical monitoring, comprehensive preconception counselling, high-dose folic acid supplementation, routine TDM and detailed foetal anomaly screening to mitigate potential teratogenic and developmental risks.^24,29,30^

More than one-third of the cohort were treated with polytherapy, contrary to national and international guidance that recommends monotherapy wherever possible.^10^ This highlights a major gap between guideline recommendations and clinical practice. Research consistently indicates that AED polytherapy is linked to higher rates of MCMs, an elevated risk of foetal growth restriction, long-term neurodevelopmental delays and an increased likelihood of stillbirth and infant mortality.^9,31^ Notably, most women receiving polytherapy were prescribed regimens that included sodium valproate, further compounding the teratogenic risk. Statistical analysis confirmed that patients on sodium valproate were significantly less likely to be managed with monotherapy. Additionally, mothers may also face increased risks, including drug interactions that can reduce the efficacy of certain AEDs or exacerbate their side effects, and central nervous system (CNS) adverse effects such as dizziness, fatigue and cognitive impairment.^9,32^ The use of multiple AEDs also raises the likelihood of poor adherence which could trigger breakthrough seizures.^9,32^ Several global pregnancy registries have confirmed the additive risks of multiple AED exposure.^21,22,24,25,26,33,34,35^

When seizures remain refractory to monotherapy, the use of polytherapy becomes essential.^9,33^ Additionally, polytherapy may be indicated in cases of drug-resistant epilepsy, where multiple AEDs are required to control life-threatening seizures.^8,9^ It is also employed in the management of multiple seizure types or epilepsy syndromes that respond more effectively to a combination of medications.^9,33^

The role of folic acid in preventing NTDs and other congenital malformations in WWE is well established.^25,33,36^ Only 70% of women in this study received the recommended high-dose folic acid (5 mg daily), leaving nearly one-third without documented use. This represents a wasted opportunity to reduce NTDs and other congenital malformations in a high-risk population. Of the women who were on sodium valproate, only 54% had record of supplemental folic acid. This is particularly concerning given that sodium valproate, carbamazepine, phenytoin and topiramate interfere with folate metabolism, considerably increasing the risk of foetal malformations.^32^ A similar trend has been reported in other studies, suggesting poor implementation of preventative strategies in epilepsy management during pregnancy.^8,9,36^ The lack of folic acid supplementation in WWE may be because of poor preconception counselling, inadequate healthcare provider awareness or reduced patient adherence. Furthermore, missed supplementation highlights the possibility of gaps at primary care level, where routine counselling and folate provision should be standardised before referral.^36^

Additionally, none of the patients received antepartum vitamin K supplementation, despite its proven benefits in preventing neonatal haemorrhagic disease in mothers taking enzyme-inducing AEDs.^34^ The Epilepsy Foundation of America (EFA) recommends vitamin K (10 – 20 mg/day) in the last month of pregnancy, yet this remains an underutilised intervention worldwide.^20^

Our study found a significantly higher caesarean section rate (73.3%) than the global average.^33^ This is in keeping with previous studies showing higher rates of operative delivery in WWE due to concerns of maternal seizures during labour, perinatal complications and foetal distress.^33^ The Epilepsy Therapy Development Project (ETDP) of EFA has also outlined a greater chance of caesarean deliveries in WWE taking AEDs.^33^ Furthermore, nearly all international guidelines have similarly warned that there is a strong possibility of preterm labour or miscarriage in WWE exists.^21,35^ The high number of caesarean deliveries in our study may be reflective of the cautionary nature of obstetric management owing to the perceived risks associated with WWE.^33^

Ten per cent of newborns were of low birth weight (< 2.5 kg), and all had been exposed to sodium valproate *in utero*; furthermore, research has also begun to establish a relationship between sodium valproate exposure and restricted foetal development.^9^ The direct association between gestational age and birth weight further supports foetal growth patterns and the reliability of the neonatal data.^9,32^ This reinforces the need for stricter sodium valproate prescribing policies, along with closer foetal growth monitoring in exposed pregnancies.^9,32^

A substantial proportion (66.7%) of WWE required hospital admission during pregnancy, with 61.7% of these being directly related to seizure management, highlighting the large burden of uncontrolled epilepsy placed upon maternal healthcare services. This is consistent with previous research by Tomson et al, which showed that WWE are at an increased risk for pregnancy complications, thus requiring frequent hospitalisation for seizure control and maternal-foetal monitoring.^23^ Sodium valproate was the most frequently prescribed AED among this cohort (64.9%) who, in addition, were more than three times likely to be admitted for seizure management, indicating poorer efficacy in maintaining seizure stability during pregnancy when compared to other AEDs.^6^ Furthermore, some patients required multiple hospitalisations, suggesting possible significant difficulties in maintaining epilepsy control, aligning with previous research on pregnancy-induced pharmacokinetic changes, such as increased renal clearance, altered protein binding and hepatic enzyme induction leading to reduced AED serum levels and increased seizure frequency.^10,22^ The Royal College of Obstetricians and Gynaecologists (RCOG) guideline published in the United Kingdom (UK) in 2016 showed an association between tonic–clonic seizures and foetal hypoxia, foetal intracranial haemorrhage and foetal loss.^15,22,24^ Multiple studies have found consistent evidence of an approximate two-fold increased risk of spontaneous abortion, stillbirth and perinatal loss in pregnant WWE.^27,33^ Although pregnancy-induced pharmacokinetic changes necessitate close TDM, only 56.7% of women had serum AED levels measured. According to Harden et al., the suboptimal use of TDM may be contributory to breakthrough seizures and subsequent hospitalisations.^24^

Ultrasound scans were performed in only 67.8% of the cases studied. This limited uptake results in considerable lost opportunities for the early detection of foetal anomalies and timely intervention in high-risk pregnancies.^6,21,22^ International guidelines advocate for ultrasonography between 11 and 13 weeks of pregnancy for NTD screening, 16-weeks for serum alpha-fetoprotein for NTD risk assessment and 18-22 weeks to assess cardiac, neurological and skeletal development.^6,21,22^ Additionally, all WWE should be offered a detailed ultrasound in line with the standards outlined by the UK National Health Service Foetal Anomaly Screening Programme, thereby ensuring comprehensive foetal monitoring during pregnancy.^12,15,37^

Most pregnant WWE are first treated at either primary or hospital-based sites, providing the potential for safer prescribing, preconceptual counselling and adequate folic acid supplementation prior to transfer to tertiary facilities.^38,39^ The high rate of sodium valproate usage, inadequate supplementation and reliance on polytherapy in this study may reflect shortcomings that typically arise earlier in the healthcare pathway.^39^ Consequently, these findings are relevant to all aspects of care and indicate the need for systemic approaches to promote coordination among multidisciplinary service providers.^27,37,38,39,40^ This is consistent with national and international guideline recommendations, which emphasise that epilepsy management in pregnancy requires action at primary, tertiary and district levels.^22,24^ Therefore, an integrative approach involving family physicians, obstetricians, neurologists, nurses, midwives and pharmacists is essential to strengthen prescribing practices, improve monitoring and surveillance, standardise supplementation regimens and ensure timely referral.^22,24^

To improve maternal and foetal health in WWE, national healthcare policies should implement stringent regulations regarding AED prescribing practices, mandate preconceptual folic acid supplementation and expand the availability and utilisation of TDM. In addition, it is essential to incorporate epilepsy-specific protocols into routine obstetric care in order to address the multifaceted risks associated with epilepsy in pregnancy.^40^ Future research should focus on long-term neurodevelopmental consequences of children exposed to AEDs *in utero*, as well as evaluate alternative treatment options to current regimens in an effort to minimise risk to the foetus while simultaneously maintaining optimal seizure control.

### Limitations

The study was conducted at a single tertiary hospital, which could limit the generalisability of the results. The analysis was also restricted to the public healthcare sector and does not reflect practices in private facilities. Some clinical records had missing data fields, which may have influenced the completeness of the analysis and led to underreporting of secondary outcomes, especially neonatal outcomes for which only half the cohort had data available. No data on planned/nplanned pregnancies were available. Furthermore, the reliance on ICD-10 codes and clinician notes introduces subjectivity and the potential for misclassification of cases. The absence of a comparison group (e.g. non-epileptic patients) could slightly limit the interpretation of the findings. Limited access to safer AEDs within the current NDoH STGs could have further hindered the ability to follow prescribed protocols.

To enhance patient safety and improve clinical outcomes, the following measures should be considered for incorporation into current treatment protocols:

- Restrict sodium valproate prescribing in women of childbearing potential (15-49-years-old) to cases where no safer alternatives exist, supported by a documented risk–benefit assessment.
- Mandating folic acid supplementation for all WWE receiving AEDs. Ensuring adequate preconception folate levels throughout pregnancy should be a fundamental aspect of epilepsy management.
- Administer antepartum vitamin K supplementation (10-20 mg/day in the final month of pregnancy) to women on enzyme-inducing AEDs to reduce neonatal haemorrhagic complications.
- Ensure routine TDM before conception, during each trimester and postpartum, with individualised dose adjustments to maintain seizure control at the lowest effective monotherapy dose.
- Minimise polytherapy whenever possible; if unavoidable, prioritise safer combinations such as lamotrigine or levetiracetam to balance efficacy with foetal safety.
- Integrate lamotrigine and levetiracetam into prescribing protocols as preferred first-line alternatives where clinically appropriate.
- Implement routine detailed anomaly ultrasounds at 18-20 weeks gestation for all pregnant WWE, ensuring timely detection of MCMs.

## Conclusion

The analysis of sodium valproate prescribing practices among women of childbearing age has revealed significant concerns regarding patient safety and adherence to established treatment guidelines. The findings indicate that a substantial proportion of patients were prescribed sodium valproate without the recommended concurrent folic acid supplementation, which leads to a critical gap in the compliance to the NDoH STGs.^23^ Given the well-documented teratogenic risks associated with sodium valproate and the potential for adverse maternal and neonatal outcomes, such prescribing patterns raise serious concerns about the optimisation of epilepsy management in this vulnerable population.^10,11,18,32^ The observed deviations from best practices, in combination with additional contributing factors, may have played a pivotal role in the increased incidence of seizure-related hospital admissions, low newborn birth weight and a higher rate of caesarean deliveries among affected patients.^1,8,41^

This study highlights several key gaps in the management of WWE during pregnancy:

- Persistent sodim valproate use despite strong teratogenic risks
- Suboptimal folic acid and vitamin K supplementation
- Elevated caesarean section rates that exceed international averages
- High rates of hospital admission driven by seizure control difficulties
- Inconsistent use of TDM and limited access to prenatal screening

Given the potential for preventable harm, it is imperative that healthcare providers, policymakers and clinical guideline committees take proactive steps to address these gaps in epilepsy management for pregnant women living with epilepsy and women of childbearing potential. Strengthening adherence to evidence-based treatment recommendations, enhancing provider education on AED teratogenicity and improving patient counselling on reproductive risks are crucial steps towards reducing adverse maternal and neonatal outcomes. By refining prescribing practices and embedding these strategies into routine care, the healthcare system can substantially improve outcomes for WWE and their children across all healthcare pathways.
